# Deep learning for sleep analysis on children with sleep-disordered breathing: Automatic detection of mouth breathing events

**DOI:** 10.3389/frsle.2023.1082996

**Published:** 2023-02-17

**Authors:** Jóna Elísabet Sturludóttir, Sigríður Sigurðardóttir, Marta Serwatko, Erna S. Arnardóttir, Harald Hrubos-Strøm, Michael Valur Clausen, Sigurveig Sigurðardóttir, María Óskarsdóttir, Anna Sigridur Islind

**Affiliations:** ^1^Department of Computer Science, Reykjavik University, Reykjavík, Iceland; ^2^Sleep Institute, Reykjavik University, Reykjavík, Iceland; ^3^Department of Engineering, Denmark's Technical University, Lyngby, Denmark; ^4^Internal Medicine Services, Landspitali University Hospital, Reykjavík, Iceland; ^5^Department of Engineering, Reykjavik University, Reykjavík, Iceland; ^6^Department of Ear, Nose and Throat Surgery, Akershus University Hospital, Lørenskog, Norway; ^7^Department of Behavioral Medicine, Faculty of Medicine, Institute of Basic Medical Sciences, University of Oslo, Oslo, Norway; ^8^Department of Immunology, Landspitali University Hospital, Reykjavík, Iceland; ^9^Faculty of Medicine, University of Iceland, Reykjavík, Iceland

**Keywords:** sleep, pediatric sleep, sleep-disordered breathing (SDB), mouth breathing, deep neural network (DNN), deep learning, convolutional neural network (CNN), machine learning

## Abstract

**Introduction:**

Sleep-disordered breathing (SDB) can range from habitual snoring to severe obstructive sleep apnea (OSA). A common characteristic of SDB in children is mouth breathing, yet it is commonly overlooked and inconsistently diagnosed. The primary aim of this study is to construct a deep learning algorithm in order to automatically detect mouth breathing events in children from polysomnography (PSG) recordings.

**Methods:**

The PSG of 20 subjects aged 10–13 years were used, 15 of which had reported snoring or presented high snoring and/or high OSA values by scoring conducted by a sleep technologist, including mouth breathing events. The separately measured mouth and nasal pressure signals from the PSG were fed through convolutional neural networks to identify mouth breathing events.

**Results:**

The finalized model presented 93.5% accuracy, 97.8% precision, 89% true positive rate, and 2% false positive rate when applied to the validation data that was set aside from the training data. The model's performance decreased when applied to a second validation data set, indicating a need for a larger training set.

**Conclusion:**

The results show the potential of deep neural networks in the analysis and classification of biological signals, and illustrates the usefulness of machine learning in sleep analysis.

## 1. Introduction

Sleep-disordered breathing (SDB) ranges from habitual snoring to severe obstructive sleep apnea (OSA) (Arnardottir et al., 2021; Óskarsdóttir et al., 2022). OSA is characterized by an obstruction in the upper airway causing it to close off resulting in repeated breathing cessations (apneas) or reduced ventilation episodes (hypopneas) during sleep (Quan et al., 1999). SDB in children is characterized by habitual snoring and upper airway resistance with OSA events (apneas and hypopneas) in more severe cases. SDB can lead to different consequences including growth impairment, neurocognitive deficits such as attention-deficit/hyperactivity disorder, and in rare cases, cardiovascular sequelae (Gottlieb et al., 2003; Li and Lee, 2009). Prior studies estimate the prevalence of OSA among children to be around 1-4% based on parent-reported symptoms from questionnaires (Lumeng and Chervin, 2008).

Humans generally utilize two breathing modes; nasal breathing and mouth breathing. Both modes have their advantages and disadvantages depending on the activity. Nasal breathing is considered the superior breathing route as it warms up the air before it enters the lungs, filters away unwanted particles, and adjusts the humidity of the incoming air (Proctor, 1977; Koutsourelakis et al., 2006). Mouth breathing, although lacking the before mentioned properties, is preferable when there is an increased need for oxygen, i.e., during certain intensities of exercise (Recinto et al., 2017). Mouth breathing during sleep, however, is a risk factor for SDB and a common characteristic of SDB in children (Young et al., 1997; Pacheco et al., 2015). In some cases, an individual might breathe partially or primarily through their mouth during parts of their sleep. In OSA patients, this can be either due to an obstruction of the nasal airway, such as rhinitis or swollen adenoids and tonsils, or simply by habit (Pacheco et al., 2015). A study by Pacheco et al. found that around 50% of the mouth breather population, aged 6–12 years, were mouth breathers by habit. Furthermore, Lavie et al. studied the effects of partial and complete mechanical obstruction of the nasal airway and found a significant increase in the number of apneas during sleep, the amount of wake after sleep onset, and the number of arousals associated with non-apneic breathing disorders with mouth breathing in sleep (Lavie et al., 1983). Habitual mouth breathers are more likely to have orofacial or craniofacial abnormalities such as long face, receding chin, open bite, and a deviated soft palate (Lam et al., 2010; Pacheco et al., 2015). Children with SDB that have undergone tonsillectomy and adenoidectomy (T&A) may continue to breathe through their mouth post operation (Pacheco et al., 2015). Myofunctional therapy can be performed to correct this breathing pattern to an extent (Pacheco et al., 2015). Performing a sleep study to confirm the ceasing of mouth breathing is therefore important.

Despite mouth breathing being a most identified characteristic of SDB in children, the symptoms are often insufficiently recognized. Mouth breathing in children is typically determined subjectively by parental report. Pacheco et al. concluded a combination of two essential tests for determining if a child is a mouth breather during daytime, the mirror test and the 3 min water retention *or* lip seal test. A mirror test is performed by placing a metal plate is below the patient's nose, against the nostrils. The patient is then to expire normally, with their mouth closed, and the area of condensation that appears on the metal plate is marked and quantified (de Pochat et al., 2011). A lip seal test is performed by completely sealing the patient's mouth with tape for 3 min or until the patient can no longer continue the test (Pacheco et al., 2015). Breathing during sleep is typically measured using a thermistor/thermocouple and a pressure transducer cannula as a part of the polysomnography (PSG). However, the thermistor/thermocouple typically measures the combined oronasal breathing and cannot quantify airflow in a reliable manner, e.g., under-detecting hypopneas (Farre et al., 1998). It is therefore also not suitable for analyzing mouth breathing alone (Lavie, 1987; Koutsourelakis et al., 2006). The pressure transducer typically only measures the nasal breathing, so mouth breathing is not detected *per se*. Separate channels are required to be able to differentiate nasal pressure from oral pressure, and consequently analyze the mouth breathing signal. This allows clinical and research studies to measure e.g., the percentage of the night a child is mouth breathing, as well as whether the child is breathing partially through the nose and mouth or only *via* the mouth (Koutsourelakis et al., 2006).

Inconsistency in the definition of mouth breathing events has caused high variability of results between studies. Some register a mouth breathing event when there is an incident of mouth breathing without the presence of nose breathing while others consider there to be an event whenever there is mouth breathing, regardless of the presence of nose breathing (Oeverland et al., 2002).

Traditionally, PSG signals are manually analyzed in 30 s portions by a sleep expert. This is an expensive and laborious process (Fischer et al., 2012; Kuna et al., 2013). Additionally, all manual processes include a human factor with potential errors and inter-scorer agreement can be low (Younes et al., 2016). Automating this process would therefore be of great interest to both decrease manual labor and increase accuracy. Deep neural network algorithms, such as convolutional neural networks (CNNs) have been used to both model and analyze biological signals and other time series data (Sabbatini, 1993; Khezri and Jahed, 2007; Belo et al., 2017; Ismail Fawaz et al., 2019). Deep learning and deep neural networks rest on the capability of automatic feature learning from data instead of hand-crafting features (Phan et al., 2018). CNN is the cornerstone of deep neural networks and has been used frequently to analyze different features in sleep recordings (Tsinalis et al., 2016; Zhang and Wu, 2017; Mikkelsen and De Vos, 2018; Phan et al., 2018).

The aim of this paper is to construct and validate a deep learning algorithm able to automatically detect mouth breathing events in children from PSG recordings.

## 2. Methods

### 2.1. Data collection

#### 2.1.1. Participants

The children are participants in the Europrevall/iFAAM study (“Integrated Approaches to Food Allergen and Allergy Risk Management”) (Keil et al., 2010; CORDIS, 2016; Grabenhenrich et al., 2020; Sigurdardottir et al., 2021). As a part of this study in Iceland, a subset of parents answered a questionnaire 2 years prior to the current data collection regarding their children.

All children with reported snoring at least three times a week or witnessed apneas at least once a week were invited to participate in this study. A subset of children that did not report any snoring or apneas was invited to participate as a control group, matched for age and sex. The study was approved by the Ethical Committee of Landspitali University Hospital and the National Bioethics Committee of Iceland (#18-206) on December 4*th* 2018. A written informed consent was obtained from a parent or legal guardian of all participants.

#### 2.1.2. Procedure

The data collection was conducted at the Children‘s Hospital in Reykjavik where each visit took approximately 2 h. Prior to the visit, the parent/legal guardian answered questionnaires online, including the Pediatric Sleep Questionnaire (PSQ) (Chervin et al., 2000) and the OSA-18 questionnaire (Sistla and Lahane, 2019). At the beginning of the visit, height and weight were measured. Then, six subtests of the Wechsler Intelligence Scale for Children (WISC-IV) (Wechsler, 2003) and the Conners‘ Continuous Performance Test (CPT) were administered (Conners, 2014). Finally, a PSG was set up for the participants to sleep with the following night at home (see details below).

#### 2.1.3. The pediatric sleep questionnaire

The PSQ assesses snoring, sleepiness, inattention and hyperactive behavior and is used to screen for SDB in children. Measures of reliability have proven reasonable-to-high for internal consistency (0.66–0.89) and test-retest reliability (0.66–0.92) (Chervin et al., 2000).

#### 2.1.4. Sleep study

All participants had a home PSG study (Nox A1, Nox Medical, Reykjavik, Iceland) set up by a sleep technologist. The study included the following sensors: electroencephalogram (EEG), electrooculogram (EOG), chin and leg electromyogram (EMG), electrocardiogram (ECG) separate nasal and mouth pressure transducers (PureFlow, Braebon Medical Corporation, Ottawa, Canada), thorax and abdomen respiratory inductance plethysmography (RIP) belts, pulse oximeter, microphone for snore analysis, electrodermal activity (EDA) and accelerometry for detection of movements and position. An additional Nox A1 device was placed on participants to assess the validity of a new, frontal EEG setup (Kainulainen et al., 2021). The mouth pressure transducer was connected to the second Nox A1 device as there is only one pressure channel on each device. PSG manual annotation was conducted by an expert sleep technologist, using the latest recommended rules by the American Academy of Sleep Medicine (version 2.5; 2018). The annotation included manual validation of automatic snore analysis by listening to the audible parts of the recording using Noxturnal version 5.1.3.20388 (Nox Medical, Reykjavik, Iceland). In the current study, additional mouth breathing annotation was conducted by an expert sleep technologist in order to create a labeled training set for a deep learning algorithm. Mouth breathing was defined as significant increase in the amplitude from the baseline of the oral pressure signal.

### 2.2. Analysis of polysomnography data

#### 2.2.1. Data extraction

European data format (EDF) files containing the PSG data were extracted for each subject from Noxturnal (Nox Medical, Reykjavik, Iceland). Additionally, manual scorings of mouth breathing events were extracted from XLSX files obtained from Noxturnal. The algorithm was then written using Python 3.8.

#### 2.2.2. The algorithm

**Figure 1:**
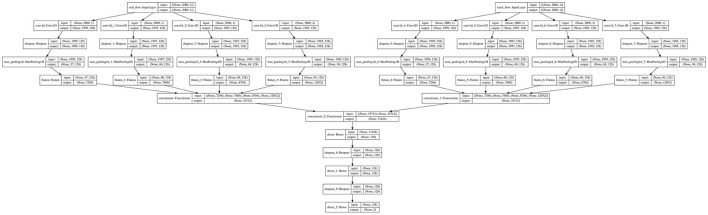
The model flowchart.

**Figure 2:**
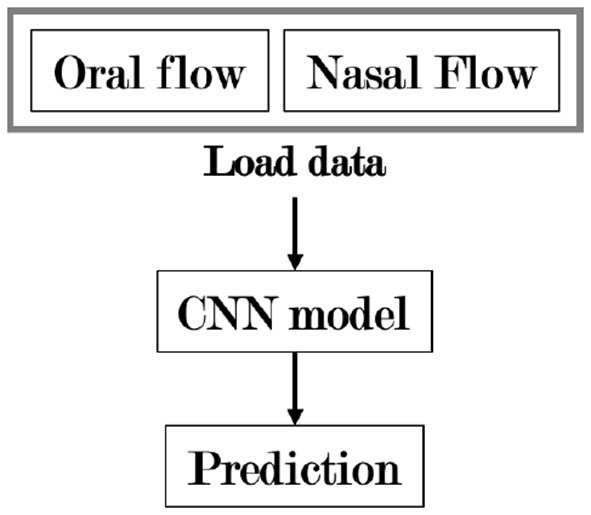
A flowchart showing the general organization of the algorithm. CNN, Convolutional neural network.

The algorithm implemented in the current study is based on the process of manually scoring mouth breathing events. The sleep technologist conducting the manual scoring for the current study also relied on the nasal breathing signal, signals of movement and other artifacts to score mouth breathing. Therefore, both the oral flow and nasal flow were used as inputs to the model. Figure 1 shows a detailed overview of the model. Figure 2 shows a flowchart explaining the general structure of the algorithm. The “Load Data” block in Figure 2 represents the main data extraction portion of the algorithm. As the two different A1 devices did not have the exact same internal clock, the data from the two devices first had to be synchronized. This was accomplished by cross-correlating the recordings using the *Activity* signal recorded on both devices, representing movement. Next, instances of mouth breathing were read from an Excel file. Then the mouth breathing and nasal breathing signals were extracted from the EDF files. Both signals were filtered using a 6th order low-pass filter, mouth breathing with a cut-off at 2 Hz, and nasal breathing with a cut-off at 1.5 Hz to filter out noise. The exact cut-off frequencies were chosen through trial and error based on the findings of Walter and Vaughn (2013). The ends of the signals that extended beyond the timeline of the other signal were trimmed off and the remaining signals normalized. Finally, the mouth breathing and nasal breathing data was cut into 10 s segments with a moving window of 5 s. Each segment was labeled with its corresponding subject number and registered in a parallel one-hot encoded target vector as either a mouth breathing event ([1,0]) or not ([0,1]).

A segment was considered a mouth breathing event if ≥ 50% of it contained mouth breathing, regardless of nasal breathing happening simultaneously. These segments were then randomized and the non-dominant class, non mouth breathing, was down-sampled to 2% of its original size. This was done to increase the balance between mouth breathing and non mouth breathing segments, as very few segments contained mouth breathing. The exact percentage was decided through trial and error. This resulted in 1941 sample segments (event: 12.6%, no event: 87.4%). The “Model” block represents the creation, training, testing, and validation of the model displayed in Figure 1. This was done using the Keras library in Python 3.8. The model was trained in 50 epochs with a batch size of 10. A total of 33% of the samples was reserved for testing, the remaining data was used to train the model with a validation split of 20%. The randomized sample sets of 10 s segments were used as inputs. As the mouth breathing signal can contain intricate patterns, a CNN was chosen to better identify landmarks specific to mouth breathing events. Convolutional layers were applied to the fine-grained breathing signals to learn low dimensional representations and correlate them with mouth breathing events. Four different filter sizes were chosen through trial and error for both signals. The filter sizes were 2, 4, 8, and 16. The layer sizes were chosen through an iterative trial and error process. The outcomes were then flattened. The output is on a one-hot-encoding format corresponding to the target vectors. Finally, training and validation accuracy, a confusion matrix, accuracy, precision, true positive rate, and false positive rate were computed.

### 2.3. Secondary model validation

To further validate the model, ten additional PSG recordings were manually scored. The oral and nasal pressure data were processed similarly to the training data. The data were filtered and split into 10 s segments that were fed to the model. The output was compared to the manual annotation. The accuracy, precision, true positive rate, and false positive rate were also computed for this analysis.

## 3. Results

### 3.1. The study cohort

**Figure 3:**
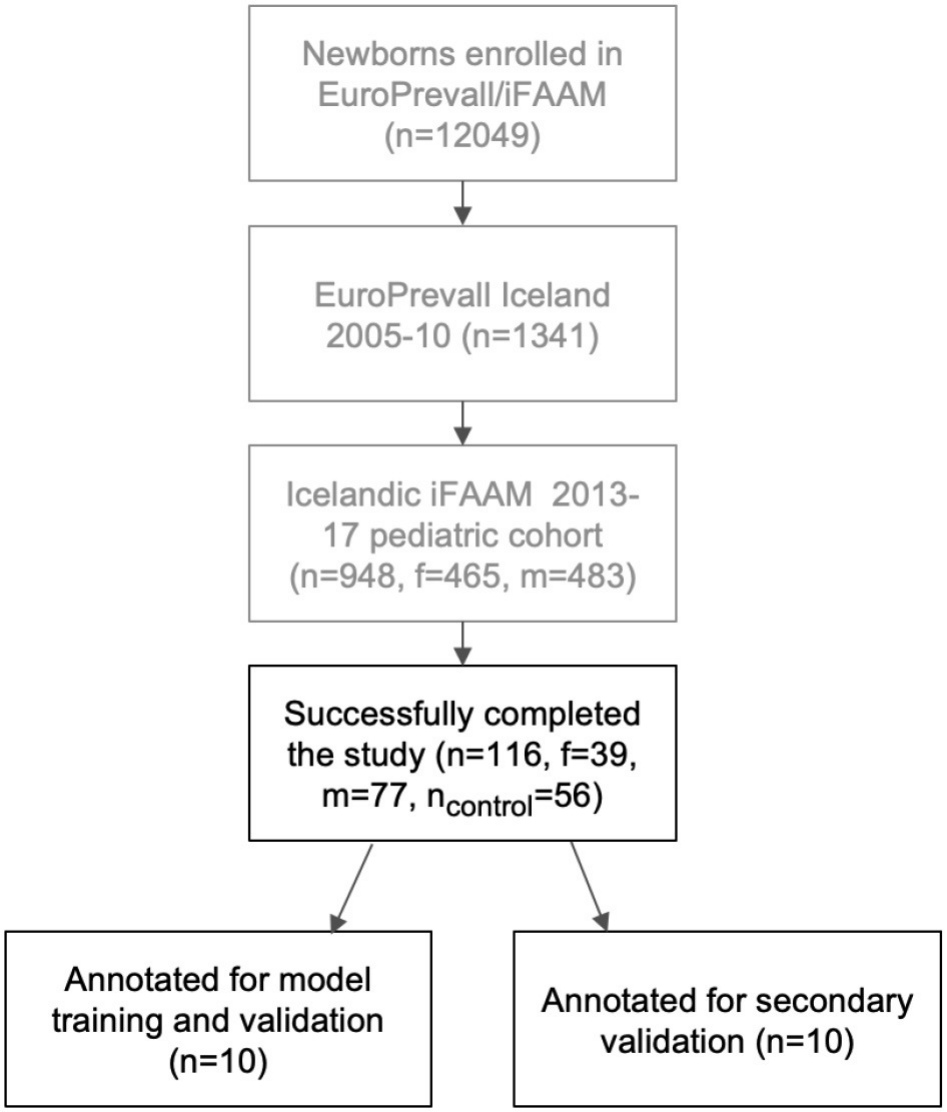
A flowchart displaying the cohort of the current study. f, female; m, male; n, number of subjects; iFAAM, Integrated Approaches to Food Allergen and Allergy Risk Management.

A total of 116 children from the iFAAM cohort successfully participated in a PSG study (60 children who had reported snoring and 56 controls). Twenty of which were manually annotated for mouth breathing events, 10 for the model training and 10 for the secondary analysis. Figure 3 shows a flowchart displaying the collection of the study cohort and Table 1 presents the demographic characteristics of the cohort. As 20 studies were annotated for mouth breathing, the resulting participants were 20 children, 6 girls and 14 boys, aged 10–13 years. The first 10 children were hand picked based on snoring and/or OSA values observed in the recordings, 5 of which were controls. The parents of the remaining 10 children had reported snoring in the OSA-18 questionnaire. The latter 10 were used in the secondary validation of the model.

**Table 1:** Demographic characteristics of the overall cohort and sub-cohorts.

**Cohort**	**Age [years]**	**M [n]**	**F [n]**	**BMI**	**Snore%**	**AHI**	**ODI**	**Arousal index**
Total (*n* = 116)	12.0 (0.8)	77	39	20.5 (3.6)	2.5 (6.3)	0.7 (0.9)	0.7 (1.1)	7.1 (2.6)
Training and validation (*n* = 10)	12.0 (0.9)	6	4	22.8 (4.2)	6.0 (9.5)	1.31 (1.3)	1.0 (1.1)	8.3 (4.4)
Secondary validation (*n* = 10)	11.0 (0.7)	8	2	21.3 (3.1)	2.8 (4.2)	0.6 (0.3)	0.5 (0.3)	7.5 (3.8)

Values are presented as mean (std) where relevant. M, Male; F, Female; BMI, Body mass index; AHI, Apnea-hypopnea index; ODI, Oxygen desaturation index.

### 3.2. The model

#### 3.2.1. Model inputs

**Figure 4:**
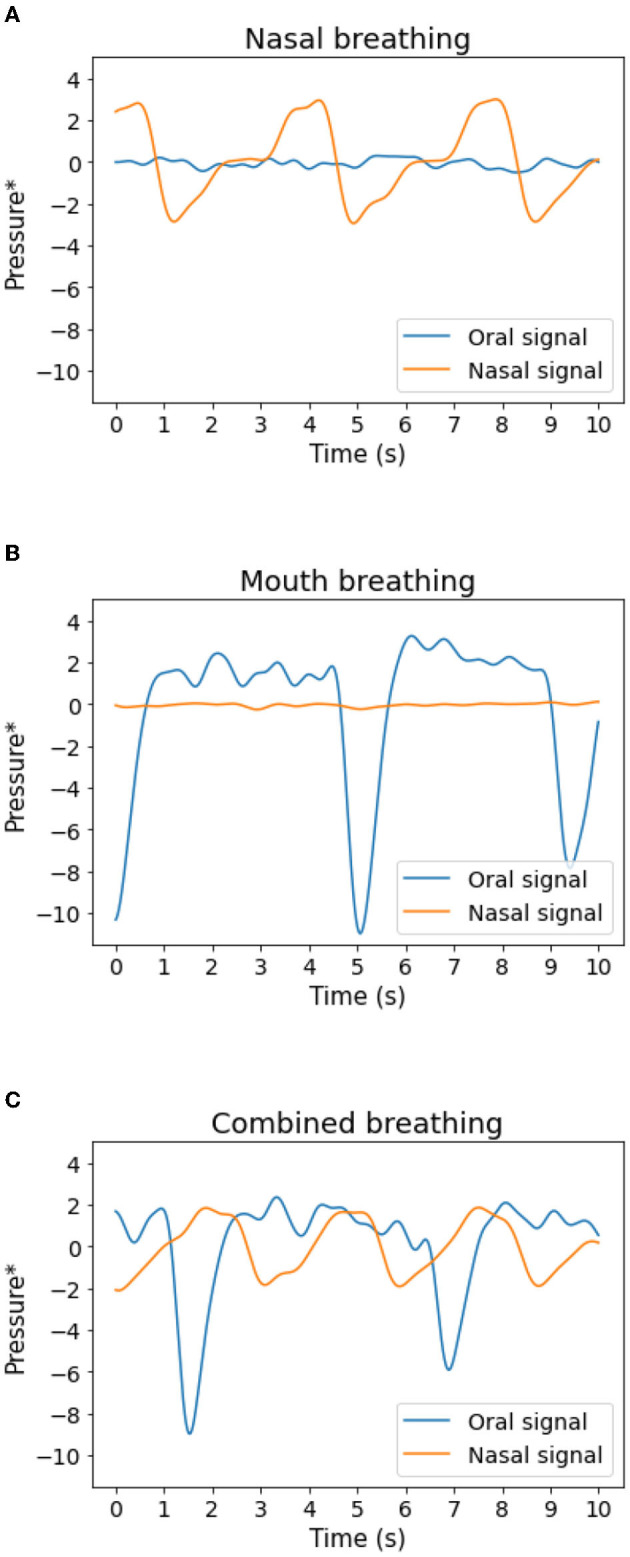
Examples of 10 s segments fed to the model. **(A)** Typical nasal breathing, **(B)** typical mouth breathing, **(C)** a mixture of mouth and nasal breathing. Blue: oral pressure. Orange: nasal pressure. *The signals have been filtered as described in Section 2.2.

Figure 4 shows examples of three typical scenarios of samples fed to the model. Blue lines represent mouth breathing, while orange lines represent nasal breathing. Figure 4A shows a typical nasal breathing segment where the nasal breathing shows its typical peaks and the mouth breathing signal is muted noise only. Figure 4B shows a typical mouth breathing segment. Here the mouth breathing follows its typical pattern, while the nasal breathing is absent as the subject is breathing exclusively through the mouth. Figure 4C shows a mixture of the two, i.e., oronasal breathing. This type of segment is classified as mouth breathing in the current study as all indications of mouth breathing were considered f interest. Note that these three examples only showcase the most typical patterns found in the data. Other variations present in the recordings, as well as artifacts, e.g., due to movement, were also presented to the model.

#### 3.2.2. Model analysis

**Figure 5:**
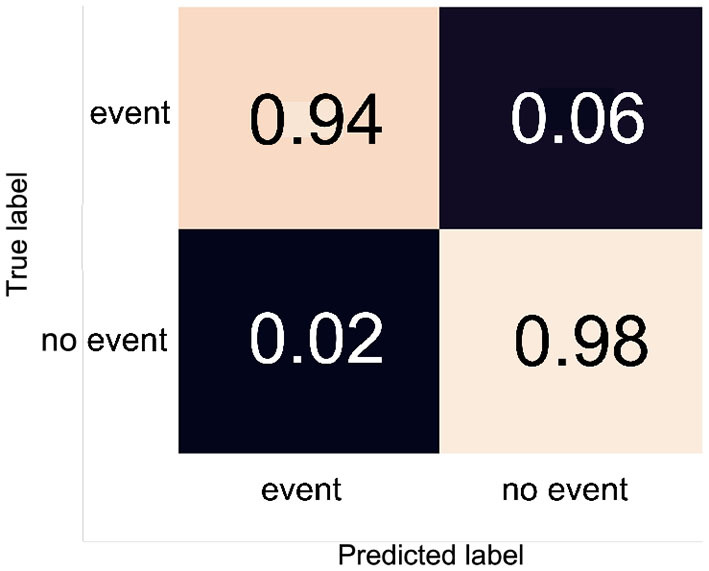
The confusion matrix obtained for the trained model. Values are proportional.

During the development of the model, a trial training run of 100 epochs showed a plateauing of the learning curve around 45 epochs. Therefore, 50 total epochs were chosen for the final model. The confusion matrix obtained from testing the model on the validation data is displayed in Figure 5. It shows that the model is highly accurate at classifying the validation data, although 6% of actual events are incorrectly classified as “no event.” Statistical parameters describing the quality of the model are presented in Table 2, along with the corresponding parameters obtained for the secondary validation data set of 10 subjects.

**Table 2:** Statistical analysis of the obtained model.

	**Validation (*n* = 10)**	**Secondary validation (*n* = 10)**
Accuracy	96.0%	97.7%
Precision	93.8%	22.1%
True positive rate	98.1%	54.4%
False positive rate	5.9%	1.8%
Sensitivity	97.9%	54.4%
Specificity	94.2%	98.2%

The performance of the model decreased significantly when met with data from new recordings. The precision of the model decreased from 93.8 to 22.1%, and the true positive rate decreased from 98.1 to 54.4%.

## 4. Discussion

Mouth breathing is a clinically valuable signal to assess during sleep. Yet, it is typically not included in a standard PSG study as it lacks a standardized measurement method and a reliable method for automation of event detection. This paper presents a method for measuring mouth breathing in pediatric sleep studies using separate nasal and oral pressure transducers, as well as a method for automating the detection of events using a deep learning algorithm. This paper is the first, to the authors knowledge, to attempt automating the detection of mouth breathing events.

The deep learning algorithm presented in this study resulted in high model accuracy when predicting on new data from the same subjects as were used to train the model. However, when predicting on data from new subjects, the model performed worse. The results of the current study imply that there is a possibility of a wider application of deep learning methods in sleep analysis in general, and for predicting mouth breathing events in sleep recordings of children in particular. By automating the analysis of signals and other observed parameters, the analytic process required for each sleep recording is reduced.

Koutsourelakis et al. (2006) researched mouth breathing and snoring in adults with SDB free of nasal obstruction. They found that OSA patients spent more time than snorers breathing only through the mouth or partially. They also found a positive correlation between the proportion of mouth and oronasal breathing and OSA severity. Our paper demonstrates the potential for clinically measuring and diagnosing mouth breathing.

Moreover, our paper utilized the pressure transducer of the A1 device to measure oral flow instead of the flawed oronasal thermistor (Lavie, 1987; Koutsourelakis et al., 2006). For diagnostic purposes, this method was found to be a functional way of measuring mouth breathing.

A limitation of the current study is the small size of the data set that represented mouth breathing. Despite many hours worth of data, sampled by overlapping a sliding window, a great majority did not represent mouth breathing events. Manual scorings of more subjects with a high number of mouth breathing events would potentially reduce the over-fitting of the model and allow the model to become increasingly familiar with a wider variety of samples, and thereby increase the usability and accuracy of the algorithm. This became prominent in the secondary validation of the model where precision and true positive rate of the model decreased significantly when met with data from never before seen recordings.

Another limitation, and a source of possible development of the current model, is the lack of precision when estimating the total time spent mouth breathing. Each segment contained 10 s, and was classified as mouth breathing only if it contained ≥50% mouth breathing. This means that each observed incident of mouth breathing should last somewhere between 5 and 10 s. A more precise method would be to look at mouth breathing as a regression problem or increase the number of classes, for example separating into 0–25%, 26–50%, 51–75%, and 76–100%. Another interesting approach would be to investigate the function of long short-term memory (LSTM) layers on longer segments. A confined attempt to use LSTM layers was made in the current study, but it did not show greater success than what could be achieved without it (data not shown). Also, as the device used only had one pressure transducer, two separate measurement devices needed to be used and the signals synchronized afterwards, as separate oral and nasal breathing assessment is not a current standard in sleep measurements. Therefore, future studies assessing whether mouth breathing can be predicted from traditional PSG sensors are needed.

## 5. Conclusion

This paper illustrates how deep learning algorithms can be applied to automatically detect mouth breathing events from PSG recordings limiting the tedious work of manual scoring. In this particular study, the focus was on learning from the mouth breathing of children with and without SDB symptoms. The final model performed very well when analyzing unknown parts of the same recordings as were used to train the model. The performance decreased when the model was presented with data from never before seen recordings. Future work could include adding a larger set of scored data with a higher number of cases with mouth breathing, to further investigate the use of LSTM layers, and to expand the use of the algorithm to other signals recorded in a PSG study.

## Data availability statement

The data analyzed in this study is subject to the following licenses/restrictions: Data is kept safe as per the ethical agreement. Requests to access these datasets should be directed to ernasifa@ru.is.

## Ethics statement

The studies involving human participants were reviewed and approved by Ethical Committee of Landspitali University Hospital and the National Bioethics Committee of Iceland (#18-206) on December 4th 2018. A written informed consent was obtained from a parent or legal guardian of all participants.

## Author contributions

JS contributed to the study design, data analysis, interpretation of the results, and wrote the first draft of the manuscript. EA, MÓ, and AI contributed to the study design, selection of data analysis methods, and writing of the manuscript. SigrS and MS contributed to the data analysis and provided insights into scoring of pediatric sleep studies. HH-S, SiguS, and MC contributed with medical expertise to the pediatric cohort, contributed to the data interpretation, and writing of the manuscript. All authors have reviewed the manuscript critically and accepted the paper in its entirety.
